# A six‐microRNA signature to predict outcomes of patients with gastric cancer

**DOI:** 10.1002/2211-5463.12593

**Published:** 2019-01-31

**Authors:** Jian Chen, Bing Hu, Wei Wang, Xiao‐jun Qian, Ben‐jie Shan, Yi‐fu He

**Affiliations:** ^1^ Department of Oncology The First Affiliated Hospital of University of Science and Technology of China Hefei China

**Keywords:** gastric cancer, microRNA, overall survival, prognosis

## Abstract

Gastric cancer (GC) is a common gastrointestinal tumor with poor prognosis. However, conventional prognostic factors cannot accurately predict the outcomes of GC patients. Therefore, there remains a need to identify novel predictive markers to improve prognosis. In this study, we obtained microRNA expression profiles of 385 GC patients from The Cancer Genome Atlas. We performed Cox regression analysis to identify overall survival‐related microRNA and then constructed a microRNA signature‐based prognostic model. The accuracy of the model was evaluated and validated through Kaplan–Meier survival analysis and time‐dependent receiver operating characteristic (ROC) curve analysis. The independent prognostic value of the model was assessed by multivariate Cox regression analysis. Enrichment analysis was performed to explore potential functions of the prognostic microRNA. Finally, a prognostic model based on a six‐microRNA (miRNA‐100, miRNA‐374a, miRNA‐509‐3, miRNA‐668, miRNA‐549, and miRNA‐653) signature was developed. Further analysis in the training, test, and complete The Cancer Genome Atlas set showed the model can distinguish between high‐risk and low‐risk patients and predict 3‐year and 5‐year survival. The six‐microRNA signature was also an independent prognostic marker, and enrichment analysis suggested that the microRNA may be involved in cell cycle and mitosis. These results demonstrated that the model based on the six‐microRNA signature can be used to accurately predict the prognosis of GC patients.

**Abbreviations**
AUC: area under the ROC curve
BP: biological process
CC: cell component
GC: gastric cancer
GO: gene ontology
HR: hazard ratio
KEGG: Kyoto Encyclopedia of Genes and Genomes
MF: molecular function
ROC: curve time‐dependent receiver operating characteristic curve
TCGA: The Cancer Genome Atlas

Gastric cancer (GC) is one of the most common gastrointestinal malignant tumors. In 2015, 1 310 000 people were diagnosed with GC around the world and 810 000 patients died because of GC. The morbidity and mortality of GC ranked 5th and 3rd among all malignant tumors, respectively 1. Due to atypical early symptoms, most patients are diagnosed with GC at an advanced stage and the median overall survival time is usually < 1 year 2, 3. On the other hand, although some patients have received radical surgery, up to 37%‐48% of them died from recurrence or metastasis 4. Therefore, the prognosis of GC is poor and it is very important and essential to improve early diagnosis and perform appropriate and individualized therapies based on prognosis.

AJCC TNM staging system is a conventional prognostic indicator. However, it is sometimes difficult to obtain an accurate stage in clinical practice for several reasons, such as < 15 lymph nodes dissection and failure to remove the tumor completely. Moreover, the AJCC staging system could not distinguish some patients at the same stage but with different survival time 5, 6. In the genomic era, the most likely explanation is the molecular heterogeneity of the patients within the same stage group. Recently, several novel molecular classification schemas of GC have been proposed according to the heterogeneous molecular characteristics 7, 8. Logically, it is also necessary and crucial to develop a novel prognostic model based on molecular characteristics to predict the outcome of patients with GC.

microRNA are a group of small noncoding RNA consisting of approximate 22 nucleotides. It has been demonstrated that one microRNA can regulate expression levels of multiple mRNA to exert its biological functions by participating in the degradation of mRNA or by inhibiting the translation of mRNA 9, 10. A number of studies have shown that microRNA are involved in proliferation 11, apoptosis 12, 13, differentiation 14, 15, invasion 16, 17, and migration 18 of GC cells. Moreover, several studies have reported that some microRNA can also affect the survival of patients with GC 19, 20. Consequently, it is feasible to construct a prognostic model based on expression profiles of microRNA.

In this study, we developed a prognostic model of GC based on six‐microRNA expression signature by using The Cancer Genome Atlas (TCGA) high‐throughput sequencing data of microRNA. The six‐microRNA expression signature was associated with overall survival and can predict 3‐ and 5‐year overall survival of patients with GC. Moreover, it was also an independent prognostic factor.

## Material and methods

### Genetic and clinical data acquisition and processing

Genetic and clinical data of patients with GC were obtained from TCGA (http://cancergenome.nih.gov/). Genetic data included microRNA and mRNA expression levels for each patient, and clinical information included age, gender, pathological stage, histological grade, survival status, and overall survival time. microRNA and mRNA expression levels were measured by log (RPM + 1) and log (FPKM + 1), respectively. microRNA that were not expressed in more than 50% of patients were removed. The patients were randomly divided into two groups which served as the training set and test set by sampling package in r program (v3.5.0, The R Foundation, Vienna, Austria), and the survival status of patients balanced between the two sets.

### Statistical analysis

Univariate and multivariate Cox regression analyses were used to identify the survival‐related microRNA in the training set. Then, the prognostic model based on the survival‐related microRNA was constructed according to Cox regression model, in which the regression coefficients represented the weights of micorRNA expression levels. The risk score of each patient was calculated by the sum of weighted expression levels of microRNA. The patients in each set were classified to the high‐risk group and low‐risk group using the median risk score in the training set as a cutoff value. Kaplan–Meier survival analyses by log‐rank test were used to compare the overall survival of patients in the two groups, and univariate Cox regression analyses were used to calculate hazard ratios (HR) between the two groups. Time‐dependent receiver operating characteristic curve (ROC curve) analyses were performed to evaluate the sensitivity and specificity of the prognostic model to predict 3‐ and 5‐year overall survival in each set by survival ROC 21 package in r program. In addition, multivariate Cox regression analyses were used to determine whether the microRNA signature was an independent prognostic marker.

### Function enrichment analysis

Since microRNA exert their biological activities through trans‐regulating mRNA, the expression correlations between microRNA and mRNA were analyzed by Pearson's correlation test. mRNA with correlation coefficients value < −0.3 and *P* < 0.05 were identified as target genes of microRNA. Subsequently, gene ontology (GO) in cell component (CC), molecular function (MF) and biological process (BP) categories, and Kyoto Encyclopedia of Genes and Genomes (KEGG) pathway enrichment analyses were performed and visualized by clusterprofiler 22 package in r program. *P* < 0.05 was considered to be significant.

## Results

### Preparation of genetic and clinical data

Genetic and clinical data of 385 patients with gastric adenocarcinoma were downloaded from the TCGA database. They were randomly assigned to the training set (*n* = 192) and test set (*n* = 193). There were no statistically significant differences in age, gender, pathological stage, histological grade, and survival status between the two sets (Table 1). After removing genes unexpressed in more than half of the samples, 566 out of total 1046 microRNA were further analyzed (Table [Link], [Link], [Link]).

**Table 1:** Clinical characteristics of patients in each dataset

	Training set (*n* = 192)	Test set (*n* = 193)	χ^2^	*P*‐value
Age (years)				
< 67	89	102	1.7697	0.183
≧67	103	88		
Gender				
Male	123	130	0.329	0.566
Female	69	63		
Histological grade				
G1/G2	69	76	0.404	0.525
G3	119	112		
Pathological stage				
I/II	82	92	0.067	0.796
III/IV	103	100		
Survival status				
Alive	118	119	0	1
Dead	74	74		

### Development of prognostic model in the training set

In the training set, by univariate Cox regression analysis, we found that the expression levels of 46 microRNA were related to the overall survival time of patients (Table S4). Subsequently, by multivariate Cox regression analysis, we found that the expression levels of six in the 46 microRNA were related to the overall survival of patients (Table 2). They were independent prognostic factors of GC patients. Among them, miRNA‐100, miRNA‐653, and miRNA‐668 were risk genes, while miRNA‐374a, miRNA‐509‐3, and miRNA‐549 were protective genes.

**Table 2:** microRNA independently associated with overall survival

	Coefficient	*P*‐value	HR	95% confidence interval
miRNA‐100	1.163	0.0212	3.199	1.193–8.576
miRNA‐374a	−1.619	0.009	0.198	0.059–0.663
miRNA‐509‐3	−1.471	0.038	0.230	0.057–0.919
miRNA‐549	−0.980	0.045	0.375	0.144–0.980
miRNA‐653	0.551	0.029	1.735	1.058–2.844
miRNA‐668	2.723	0.021	15.224	1.512–153.341

To construct a prognostic model, multivariate Cox regression analysis was performed on the six microRNA with independent prognostic value, and the weight of each microRNA expression level in the predictive model was obtained according to the regression coefficient. The risk score was defined as follows: Risk score = (0.336*expression level of miRNA‐100) + (−0.777*expression level of miRNA‐374a) + (−0.578*expression level of miRNA‐509‐3) + (−0.487*expression level of miRNA‐549) +  (0.618*expression level of miRNA‐653) + (1.223*expression level of miRNA‐668).

**Figure 1:**
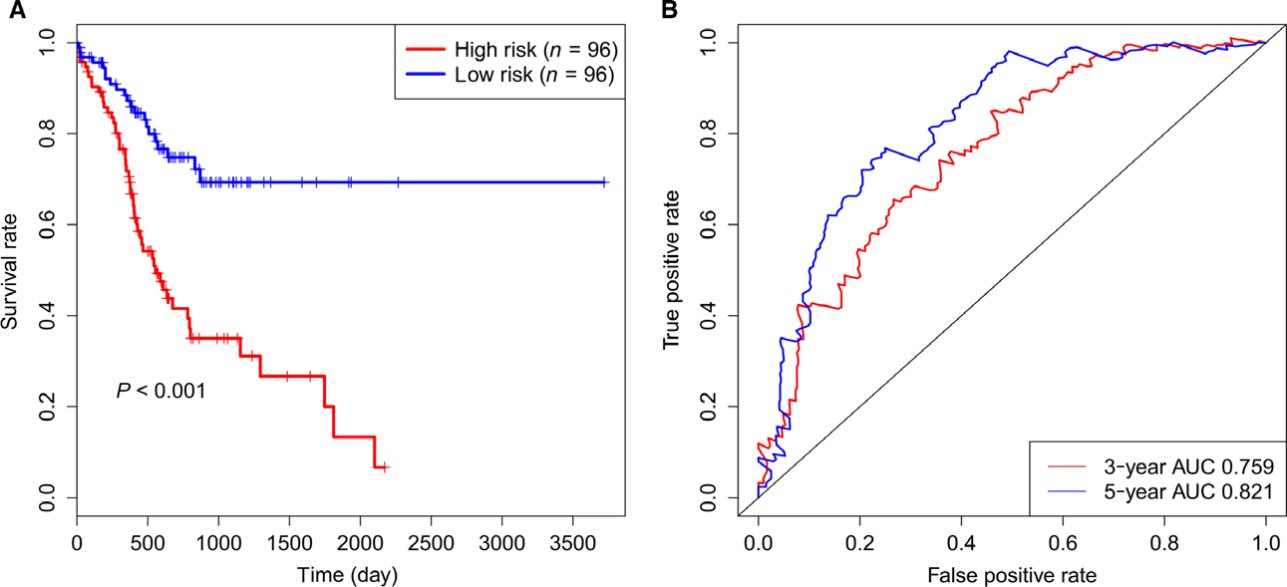
The prognostic performance of the six‐microRNA signature in the training set. (A) Kaplan–Meier survival analysis by log‐rank test between the high‐risk group and low‐risk group in the training set. (B) Time‐dependent ROC analysis for the six‐microRNA signature to predict 3‐ and 5‐year survival.

Based on this model, the risk score of each patient was calculated and there were 96 patients in the high‐risk group and 96 patients in the low‐risk group in the training set using the median risk score of patients in the training set as cutoff value. Kaplan–Meier survival analysis by log‐rank test demonstrated that there was a significant difference between the two groups. Patients in the low‐risk group tended to have longer overall survival time than those in the high‐risk group (*P* < 0.001, Fig. 1A). The univariate Cox regression analysis indicated that the HR of high‐risk group versus low‐risk group was 3.154 (95% CI: 1.899–5.24, *P* < 0.001, Table 3). Furthermore, time‐dependent ROC analysis of the six‐microRNA signature showed that the area under the ROC curve (AUC) reached 0.759 and 0.821 to predict 3‐ and 5‐year survival (Fig. 1B). Therefore, the six‐microRNA signature‐based model can predict the prognosis of patients.

**Table 3:** Univariate and multivariate analyses of clinical characteristics and the six‐microRNA signature

	Univariate analysis			Multivariate analysis		
	HR	95% CI	*P*‐value	HR	95% CI	*P*‐value
Training set						
Age (< 67/≧ 67 years)	1.372	0.861–2.186	0.184	1.63	1.012–2.628	0.045
Gender (male/female)	0.832	0.513–1.348	0.455	0.913	0.559–1.490	0.715
Histological grade (G1, G2/G3)	1.973	1.157–3.365	0.013	1.696	0.953–2.983	0.058
Pathological stage (I, II/III, IV)	1.927	1.174–3.163	0.01	1.711	1.032–2.839	0.037
Six‐microRNA signature (low risk/high risk)	3.154	1.899–5.24	< 0.001	2.682	1.597–4.504	< 0.001
Test set						
Age (< 67/≧ 67 years)	1.344	0.844–2.14	0.212	1.792	1.087–2.953	0.022
Gender (male/female)	0.76	0.454–1.272	0.296	0.677	0.386–1.189	0.175
Histological grade (G1, G2/G3)	1.04	0.648–1.67	0.871	0.946	0.573–1.563	0.83
Pathological stage (I, II/III, IV)	1.958	1.208–3.174	0.006	1.819	1.096–3.019	0.021
Six‐microRNA signature (low risk/high risk)	1.699	1.07–2.698	0.025	1.702	1.039–2.787	0.035
Entire TCGA set						
Age (< 67/≧ 67 years)	1.324	0.957–1.831	0.09	1.645	1.176–2.302	0.004
Gender (male/female)	0.807	0.568–1.146	0.231	0.768	0.535–1.102	0.152
Histological grade (G1, G2/G3)	1.406	0.993–1.991	0.055	1.232	0.86–1.765	0.256
Pathological stage (I, II/III, IV)	1.94	1.376–2.734	< 0.001	1.724	1.211–2.455	0.003
Six‐microRNA signature (low risk/high risk)	2.3	1.646–3.216	< 0.001	2.12	1.496–3.005	< 0.001

### Validation of the prognostic model in testing and entire TCGA set

**Figure 2:**
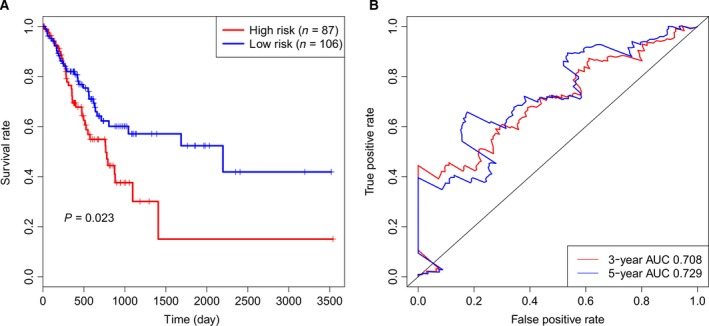
The prognostic performance of the six‐microRNA signature in the test set. (A) Kaplan–Meier survival analysis by log‐rank test between the high‐risk group and low‐risk group in the test set. (B) Time‐dependent ROC analysis for the six‐microRNA signature to predict 3‐ and 5‐year survival.

To assess the predictive value of this model, we further validated the six‐microRNA signature in the test set. By using the same risk score calculation method, the 193 patients in the test set were divided into the high‐risk group (*n* = 87) and low‐risk group (*n* = 106) according to the same cutoff value as used in the training set. The result of Kaplan–Meier survival analysis was consistent with that in the training set. The patients in the low‐risk group tend to have longer overall survival time than those in the high‐risk group (*P* = 0.023, Fig. 2A). The HR of the high‐risk group versus the low‐risk group was 1.699 (95% CI: 1.07–2.698, *P* = 0.025, Table 3) according to univariate Cox regression analysis. The AUC in time‐dependent ROC analysis was 0.708 at 3‐year survival and 0.729 at 5‐year survival (Fig. 2B). These results showed that the model also performed well in the test set.

**Figure 3:**
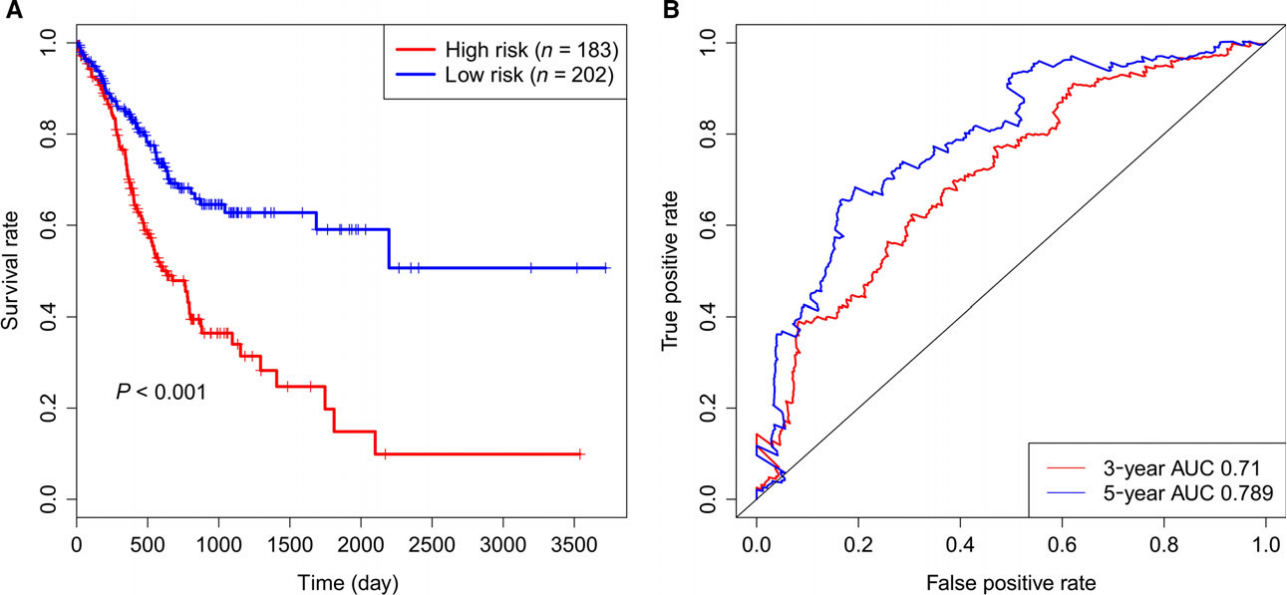
The prognostic performance of the six‐microRNA signature in the entire TCGA set. (A) Kaplan–Meier survival analysis by log‐rank test between the high‐risk group and low‐risk group in the entire TCGA set. (B) Time‐dependent ROC analysis for the six‐microRNA signature to predict 3‐ and 5‐year survival.

To further verify the robustness of the prognostic model, the six‐microRNA signature was tested in the entire TCGA set. By using the same risk cutoff criteria as above, the patients in the entire TCGA set were classified into the high‐risk group (*n* = 183) and low‐risk group (*n* = 202). Similar result of Kaplan–Meier survival analysis by log‐rank test was observed. The patients in the low‐risk group tended to have better overall survival than those in the high‐risk group (*P* < 0.001, Fig. 3A). The univariate Cox regression analysis showed that the HR of the high‐risk group versus the low‐risk group was 2.3 (95% CI: 1.646–3.216, *P* < 0.001, Table 3). Time‐dependent ROC analyses illustrated that the AUC of the prognostic model to predict 3‐ and 5‐year survival was 0.71 and 0.789 (Fig. 3B). These analyses on the entire TCGA set confirmed the robustness of the six‐microRNA signature.

### Assessment of independence value of the six‐microRNA signature

**Figure 4:**
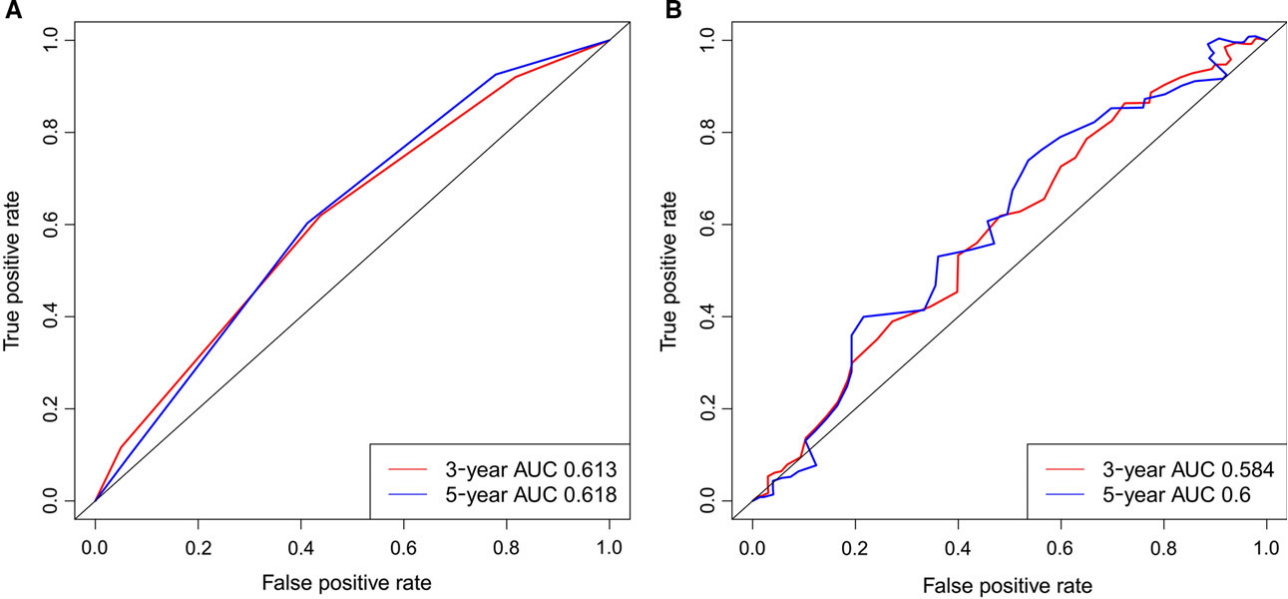
Time‐dependent ROC analysis for pathological stage and age to predict 3‐ and 5‐year survival in the entire TCGA set. (A) Pathological stage. (B) Age.

To assess the independent prognostic value of six‐microRNA signature, multivariate Cox regression analyses were performed. The consistent results in the training, test, and entire TCGA set showed that pathological stage, age, and the six‐microRNA signature were independent prognostic markers of patients with GC. The HR of high‐risk group versus low‐risk group was 2.682 (95% CI: 1.597‐4–504) in the training set, 1.702 (95% CI: 1.039–2.787) in the test set, and 2.120 (95% CI: 1.496–3.005) in the entire TCGA set. However, time‐dependent ROC analysis of pathological stage and age to predict 3‐ and 5‐year survival revealed that the AUCs were < 0.7 (Fig. 4A,B). These results demonstrated that the six‐microRNA signature was an independent prognostic marker of GC patients and superior to pathological stage and age.

### Function pathway enrichment analysis of the six microRNA

**Figure 5:**
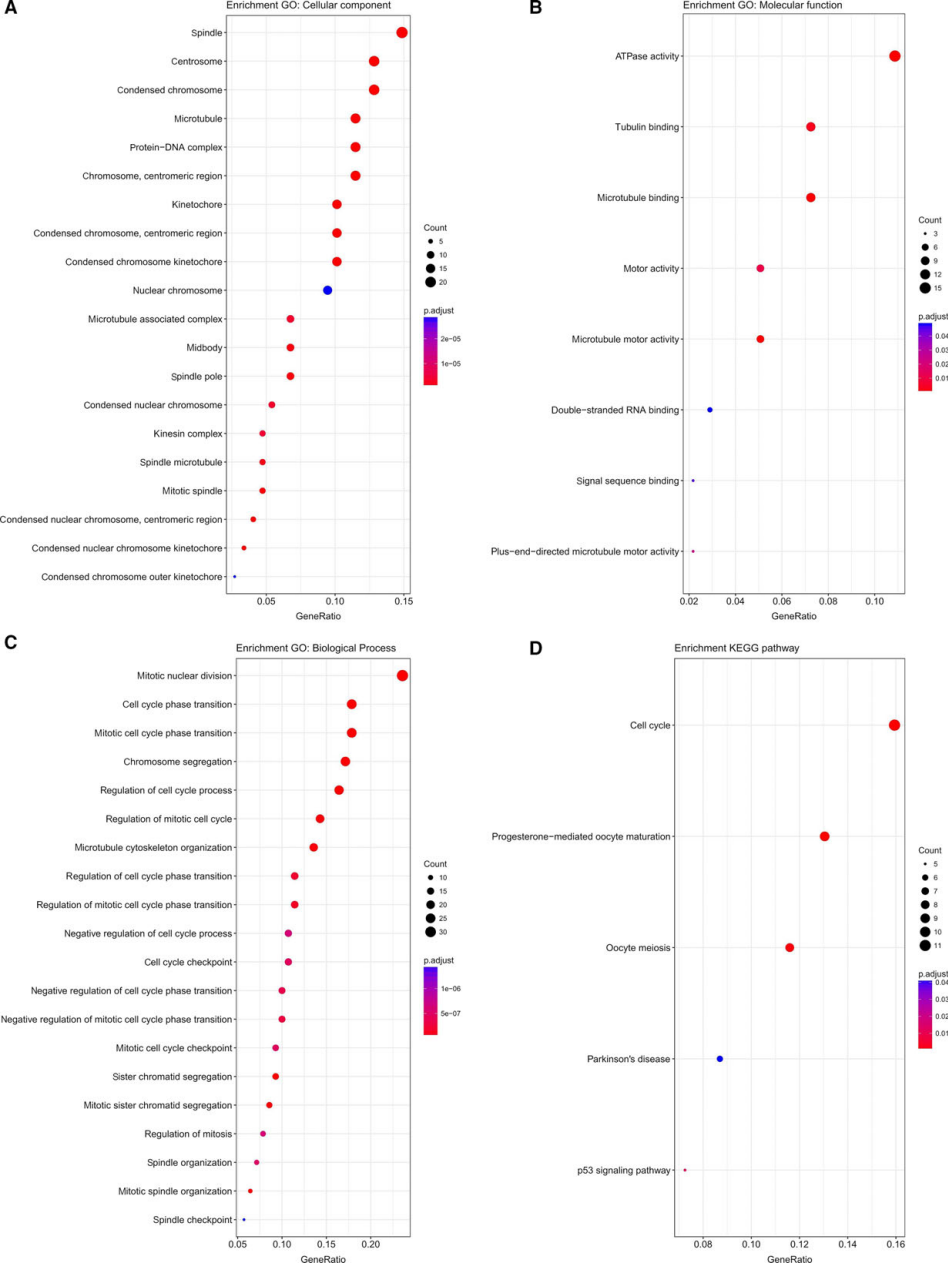
GO and KEGG pathway enrichment analysis. (A) Top 20 significantly enriched cellular component GO annotations. (B) Significantly enriched BP GO annotations. (C) Top 20 significantly enriched MF GO annotations. (D) Significantly enriched KEGG pathways.

To explore potential functions of these six microRNA, 978 co‐expressed mRNA, which may be the target genes of the microRNA, were identified by Pearson's correlation test. GO enrichment analysis of the co‐expressed mRNA suggested that chromosome, centromeric region, ATPase activity, and mitotic nuclear division were the most significantly enriched CC, MF, and BP categories (Fig. 5A–C, Table S5). KEGG pathway enrichment analysis indicated that cell cycle was the most significantly enriched pathway (Fig. 5D, Table S6). In addition, these mRNA also functioned as microtubule binding, tubulin binding, etc., which have been proved to be related to cell proliferation. They were also involved in some cancer‐related biologic processes or signal pathways such as cell cycle phase transition, cell cycle checkpoint, regulation of cell division, and p53 signal pathway.

## Discussion

In the present study, we identified six survival‐related microRNA in patients with GC by Cox regression model and proposed a six‐microRNA signature‐based prognostic model. The model can distinguish the patients of GC with poor and good prognosis, and the ROC curve analysis showed that the AUC of the model to predict 3‐ or 5‐year overall survival was > 0.7. In addition, according to the multivariate Cox regression analysis, the six‐microRNA signature was also an independent prognostic marker. These results, which were validated in the training set, test set, and entire TCGA set, illustrated that the model based on six‐microRNA signature was robust to predict the outcomes of patients with GC.

There have been several similar studies which developed prognostic models of GC depending on molecular profiles. Tow studies have constructed prognostic models based on the mRNA signature. However, both the sample sizes were relatively small 23, 24. Another study conducted by Wang *et al*. 25 built a model based on a nine‐mRNA signature to predict the prognosis of GC patients. It can distinguish patients with high risk or low risk in a cohort but cannot predict the prognosis of a single patient, because the evaluation method was based on median gene expression levels of the cohort. Recently, noncoding RNA were also used to construct prognostic models. Miao *et al*. 26 proposed a four lncRNA‐based prognostic model of GC. However, the AUC of time‐dependent ROC curve to predict 5‐year overall survival was < 0.7. Another study 27 developed a microRNA‐based model, but it did not evaluate the prognostic value on predicting 3‐ and 5‐year survival. Compared with these studies, the model in the current study can distinguish the patients with poor or good prognosis, and it also performed well in predicting 3‐ and 5‐year survival.

In our study, six microRNA were identified to be associated with overall survival. The enrichment analyses revealed that the target mRNA of them took part in process of cell cycle, mitosis, p53 signal pathway, etc. These results can explain why the six microRNA were related to the prognosis of patients. On the other hand, most of the microRNA have been found to be related to tumors. Among these microRNA, miRNA‐100 and miRNA‐374a were the most frequently studied microRNA. Nevertheless, controversial results about their roles in tumors have been reported. miRNA‐100 was upregulated in patients with diffuse‐type GC and related to the depth of invasion, lymph node metastasis, and stage 28. On the contrary, another study showed that miRNA‐100 could promote apoptosis of GC cell through Notch‐apoptosis pathway and improve the sensitivity of GC cells to chemotherapy 29. miRNA‐374a could promote proliferation, migration, and invasion of GC cells through downregulating SRCIN1 while inhibit proliferation, invasion, migration, and intrahepatic metastasis of colon cancer cells by targeting CCND1 30, 31. In our study, miRNA‐100 was a risk microRNA and miRNA‐374a was a protective microRNA. These inconsistent results may be due to the different tumor types or microenvironments such as *in vitro* and *in vivo*. miRNA‐509‐3 has been previously identified as a tumor suppressor gene in lung cancer 32, ovarian cancer 33, hepatoma 34, leukemia 35, renal cell carcinoma 36, and GC 37. It was also an independent prognostic biomarker in GC patients. These findings were consistent with ours. miRNA‐668 might play a role of oncogene 38 and could be associated with radioresistance in breast cancer 39. In our study, similar results were found and showed that miRNA‐668 was a risk gene in GC. To date, there have been no direct studies focusing on the relationships between miRNA‐549 or miRNA‐653 and tumors. However, our study showed that miRNA‐549 was a protective microRNA and miRNA‐653 was a risk microRNA, which deserve further study.

In summary, our study identified six survival‐related microRNA (miRNA‐100, miRNA‐374a, miRNA‐509‐3, miRNA‐668, miRNA‐549, and miRNA‐653) in GC patients and developed a prognostic prediction model. The model can be utilized to predict the risk of death and 3‐ and 5‐year overall survival for patients with GC. Moreover, the six‐microRNA signature of the model was also a novel independent molecular prognostic biomarker. These results will contribute to individualized therapies for GC patients.

## Author contributions

Y‐fH and JC conceived and designed the experiments. BH reviewed drafts of the paper. B‐jS and WW performed the experiments and analyzed the data. X‐jQ prepared figures and/or tables.

## Conflicts of interest

The authors declare no conflict of interest.

## Supporting information


**Table S1.** Training set.

**Table S2.** Test set.

**Table S3.** Clinical information.

**Table S4.** Survival‐related microRNA according to univariate Cox regression analysis.

**Table S5**. GO enrichment analysis of the co‐expressed mRNA.

**Table S6**. KEGG pathwy enrichment analysis of the co‐expressed mRNA.

## References

[1] Fitzmaurice C , Allen C , Barber RM , Barregard L , Bhutta ZA , Brenner H , Dicker DJ , Chimed‐Orchir O , Dandona R , Dandona L *et al* (2017) Global, regional, and national cancer incidence, mortality, years of life lost, years lived with disability, and disability‐adjusted life‐years for 32 cancer groups, 1990 to 2015: a systematic analysis for the global burden of disease study. JAMA Oncol 3, 524–548. 27918777 10.1001/jamaoncol.2016.5688 PMC6103527

[2] Hohenberger P and Gretschel S (2003) Gastric cancer. Lancet 362, 305–315. 12892963 10.1016/s0140-6736(03)13975-x

[3] Ychou M , Boige V , Pignon JP , Conroy T , Bouche O , Lebreton G , Ducourtieux M , Bedenne L , Fabre JM , Saint‐Aubert B *et al* (2011) Perioperative chemotherapy compared with surgery alone for resectable gastroesophageal adenocarcinoma: an FNCLCC and FFCD multicenter phase III trial. J Clin Oncol 29, 1715–1721. 21444866 10.1200/JCO.2010.33.0597

[4] Songun I , Putter H , Kranenbarg EM , Sasako M and van de Velde CJ (2010) Surgical treatment of gastric cancer: 15‐year follow‐up results of the randomised nationwide Dutch D1D2 trial. Lancet Oncol 11, 439–449. 20409751 10.1016/S1470-2045(10)70070-X

[5] Rocken C and Behrens HM (2015) Validating the prognostic and discriminating value of the TNM‐classification for gastric cancer ‐ a critical appraisal. Eur J Cancer 51, 577–586. 25682192 10.1016/j.ejca.2015.01.055

[6] Liu JY , Peng CW , Yang XJ , Huang CQ and Li Y (2018) The prognosis role of AJCC/UICC 8(th) edition staging system in gastric cancer, a retrospective analysis. Am J Transl Res 10, 292–303. 29423014 PMC5801367

[7] Lei Z , Tan IB , Das K , Deng N , Zouridis H , Pattison S , Chua C , Feng Z , Guan YK , Ooi CH *et al* (2013) Identification of molecular subtypes of gastric cancer with different responses to PI3‐kinase inhibitors and 5‐fluorouracil. Gastroenterology 145, 554–565. 23684942 10.1053/j.gastro.2013.05.010

[8] Cancer Genome Atlas Research Network (2014) Comprehensive molecular characterization of gastric adenocarcinoma. Nature 513, 202–229. 25079317 10.1038/nature13480 PMC4170219

[9] Filipowicz W , Bhattacharyya SN and Sonenberg N (2008) Mechanisms of post‐transcriptional regulation by microRNAs: are the answers in sight? Nat Rev Genet 9, 102–114. 18197166 10.1038/nrg2290

[10] Carthew RW and Sontheimer EJ (2009) Origins and mechanisms of miRNAs and siRNAs. Cell 136, 642–655. 19239886 10.1016/j.cell.2009.01.035 PMC2675692

[11] Wu Q , Jin H , Yang Z , Luo G , Lu Y , Li K , Ren G , Su T , Pan Y , Feng B *et al* (2010) MiR‐150 promotes gastric cancer proliferation by negatively regulating the pro‐apoptotic gene EGR2. Biochem Biophys Res Comm 392, 340–345. 20067763 10.1016/j.bbrc.2009.12.182

[12] Zhang X , Nie Y , Du Y , Cao J , Shen B and Li Y (2012) MicroRNA‐181a promotes gastric cancer by negatively regulating tumor suppressor KLF6. Tumour Biol 33, 158915–158997. 10.1007/s13277-012-0414-3 22581522

[13] Li J , Guo Y , Liang X , Sun M , Wang G , De W and Wu W (2012) MicroRNA‐223 functions as an oncogene in human gastric cancer by targeting FBXW7/hCdc4. J Cancer Res Clin Oncol 138, 763–7674. 22270966 10.1007/s00432-012-1154-x PMC11824240

[14] Zhang BG , Li JF , Yu BQ , Zhu ZG , Liu BY and Yan M (2012) microRNA‐21 promotes tumor proliferation and invasion in gastric cancer by targeting PTEN. Oncol Rep 27, 1019–1026. 22267008 10.3892/or.2012.1645 PMC3583594

[15] Zhu Y , Jiang Q , Lou X , Ji X , Wen Z , Wu J , Tao H , Jiang T , He W , Wang C *et al* (2012) MicroRNAs up‐regulated by CagA of Helicobacter pylori induce intestinal metaplasia of gastric epithelial cells. PLoS ONE 7, e35147. 22536353 10.1371/journal.pone.0035147 PMC3335061

[16] Yang Q , Jie Z , Cao H , Greenlee AR , Yang C , Zou F and Jiang Y (2011) Low‐level expression of let‐7a in gastric cancer and its involvement in tumorigenesis by targeting RAB40C. Carcinogenesis 32, 713–722. 21349817 10.1093/carcin/bgr035

[17] Tie J , Pan Y , Zhao L , Wu K , Liu J , Sun S , Guo X , Wang B , Gang Y , Zhang Y *et al* (2010) MiR‐218 inhibits invasion and metastasis of gastric cancer by targeting the Robo1 receptor. PLoS Genet 6, e1000879. 20300657 10.1371/journal.pgen.1000879 PMC2837402

[18] Zhao X , Dou W , He L , Liang S , Tie J , Liu C , Li T , Lu Y , Mo P , Shi Y *et al* (2013) MicroRNA‐7 functions as an anti‐metastatic microRNA in gastric cancer by targeting insulin‐like growth factor‐1 receptor. Oncogene 32, 1363–1372. 22614005 10.1038/onc.2012.156

[19] Huo Q (2017) Analysis of expression profile of miRNA in stomach adenocarcinoma. J BUON 22, 1154–1159. 29135097

[20] Xiong X , Ren HZ , Li MH , Mei JH , Wen JF and Zheng CL (2011) Down‐regulated miRNA‐214 induces a cell cycle G1 arrest in gastric cancer cells by up‐regulating the PTEN protein. Pathol Oncol Res 17, 931–937. 21688200 10.1007/s12253-011-9406-7

[21] Heagerty PJ , Lumley T and Pepe MS (2000) Time‐dependent ROC curves for censored survival data and a diagnostic marker. Biometrics 56, 337–344. 10877287 10.1111/j.0006-341x.2000.00337.x

[22] Yu G , Wang LG , Han Y and He QY (2012) clusterProfiler: an R package for comparing biological themes among gene clusters. OMICS 16, 284–287. 22455463 10.1089/omi.2011.0118 PMC3339379

[23] Xu ZY , Chen JS and Shu YQ (2010) Gene expression profile towards the prediction of patient survival of gastric cancer. Biomed Pharmacother 64, 133–139. 20005068 10.1016/j.biopha.2009.06.021

[24] Chen CN , Lin JJ , Chen JJ , Lee PH , Yang CY , Kuo ML , Chang KJ and Hsieh FJ (2005) Gene expression profile predicts patient survival of gastric cancer after surgical resection. J Clin Oncol 23, 7286–7295. 16145069 10.1200/JCO.2004.00.2253

[25] Wang Z , Chen G , Wang Q , Lu W and Xu M (2017) Identification and validation of a prognostic 9‐genes expression signature for gastric cancer. Oncotarget 8, 73826–73836. 29088749 10.18632/oncotarget.17764 PMC5650304

[26] Miao Y , Sui J , Xu SY , Liang GY , Pu YP and Yin LH (2017) Comprehensive analysis of a novel four‐lncRNA signature as a prognostic biomarker for human gastric cancer. Oncotarget 8, 75007–75024. 29088841 10.18632/oncotarget.20496 PMC5650396

[27] Li X , Zhang Y , Zhang Y , Ding J , Wu K and Fan D (2010) Survival prediction of gastric cancer by a seven‐microRNA signature. Gut 59, 579–585. 19951901 10.1136/gut.2008.175497

[28] Ueda T , Volinia S , Okumura H , Shimizu M , Taccioli C , Rossi S , Alder H , Liu CG , Oue N , Yasui W *et al* (2010) Relation between microRNA expression and progression and prognosis of gastric cancer: a microRNA expression analysis. Lancet Oncol 11, 136–146. 20022810 10.1016/S1470-2045(09)70343-2 PMC4299826

[29] Yang G , Gong Y , Wang Q , Wang Y and Zhang X (2015) The role of miR‐100‐mediated Notch pathway in apoptosis of gastric tumor cells. Cell Signal 27, 1087–1101. 25703026 10.1016/j.cellsig.2015.02.013

[30] Xu X , Wang W , Su N , Zhu X , Yao J , Gao W , Hu Z and Sun Y (2015) miR‐374a promotes cell proliferation, migration and invasion by targeting SRCIN1 in gastric cancer. FEBS Lett 589, 407–413. 25554419 10.1016/j.febslet.2014.12.027

[31] Chen Y , Jiang J , Zhao M , Luo X , Liang Z , Zhen Y , Fu Q , Deng X , Lin X , Li L *et al* (2016) microRNA‐374a suppresses colon cancer progression by directly reducing CCND1 to inactivate the PI3K/AKT pathway. Oncotarget 7, 41306–41319. 27191497 10.18632/oncotarget.9320 PMC5173061

[32] Wang XH , Lu Y , Liang JJ , Cao JX , Jin YQ , An GS , Ni JH , Jia HT and Li SY (2016) MiR‐509‐3‐5p causes aberrant mitosis and anti‐proliferative effect by suppression of PLK1 in human lung cancer A549 cells. Biochem Biophys Res Comm 478, 676–682. 27498003 10.1016/j.bbrc.2016.08.006

[33] Pan Y , Robertson G , Pedersen L , Lim E , Hernandez‐Herrera A , Rowat AC , Patil SL , Chan CK , Wen Y , Zhang X *et al* (2016) miR‐509‐3p is clinically significant and strongly attenuates cellular migration and multi‐cellular spheroids in ovarian cancer. Oncotarget 7, 25930–25948. 27036018 10.18632/oncotarget.8412 PMC5041955

[34] Wang Y , Cui M , Cai X , Sun B , Liu F , Zhang X and Ye L (2014) The oncoprotein HBXIP up‐regulates SCG3 through modulating E2F1 and miR‐509‐3p in hepatoma cells. Cancer Lett 352, 169–178. 24882622 10.1016/j.canlet.2014.05.007

[35] Tan YS , Kim M , Kingsbury TJ , Civin CI and Cheng WC (2014) Regulation of RAB5C is important for the growth inhibitory effects of MiR‐509 in human precursor‐B acute lymphoblastic leukemia. PLoS ONE 9, e111777. 25368993 10.1371/journal.pone.0111777 PMC4219775

[36] Zhang WB , Pan ZQ , Yang QS and Zheng XM (2013) Tumor suppressive miR‐509‐5p contributes to cell migration, proliferation and antiapoptosis in renal cell carcinoma. Ir J Med Sci 182, 621–627. 23619562 10.1007/s11845-013-0941-y

[37] Zhang J , Zhu Z , Sheng J , Yu Z , Yao B , Huang K , Zhou L , Qiu Z and Huang C (2017) miR‐509‐3‐5P inhibits the invasion and lymphatic metastasis by targeting PODXL and serves as a novel prognostic indicator for gastric cancer. Oncotarget 8, 34867–34883. 28432273 10.18632/oncotarget.16802 PMC5471018

[38] Sevinc ED , Cecener G , Ak S , Tunca B , Egeli U , Gokgoz S , Tolunay S and Tasdelen I (2016) Expression and clinical significance of miRNAs that may be associated with the FHIT gene in breast cancer. Gene 590, 278–284. 27236032 10.1016/j.gene.2016.05.033

[39] Luo M , Ding L , Li Q and Yao H (2017) miR‐668 enhances the radioresistance of human breast cancer cell by targeting IkappaBalpha. Breast Cancer 24, 673–682. 28138801 10.1007/s12282-017-0756-1

